# Rays and the Cancer Problem

**Published:** 1919-05-10

**Authors:** 


### RAYS AND THE CANCER PROBLEM.

### Their Value and Limitations.

Held in many quarters to be most disappointing in its results, the application of radium and $x$-rays in the treatment of cancer has lately fallen, if not into disuse, into relative obscurity. There are still a number of surgeons who advise and employ these agencies in typically inoperable cases, but it is doubtful whether the full possibilities are generally appreciated. Although the haphazard use of radio-activity is certainly to be discouraged, the indications for its application are neither so intricate nor so specialised that they cannot become common knowledge among the profession.

Its advocates do not claim that the method can ever supersede surgery, but only that it is a valuable adjunct to this art, helping to prevent recurrence after operation, sometimes rendering inoperable cases operable, proving itself one of the best palliatives we have, and bringing about, in some locationally favourable instances at least, an apparent cure. In any case it is an established fact that radium emanation and hard $x$-rays have some power to prevent proliferation of the cancer cell. Also it is probable that many of the past "failures" are due to the treatment having been set an impossible task with tumours, the outlying portions of which it was obviously unable to influence.

### A Physical Outline.

The lines along which this form of treatment may develop are very directly controlled by physical laws and discovery, and one may devote a few words to indicate roughly our present conception of them. The emanation emitted by radium and radio-active bodies consists of rays of three distinct types. The "alpha" rays, or more correctly particles, affect a photographic plate, cause many bodies brilliantly to fluoresce, and have strong ionising but very poor penetrating power. In fact in passage through air they are readily stopped by the gaseous molecules which they encounter in their flight. Rutherford has shown them to consist of positively charged particles of helium. The "beta" rays consist, according to Meyer and Schweidler, of negatively charged particles. Compared with the "alpha," they are relatively inactive photographically and in ionising power, but are capable of penetrating a considerable thickness of solid or liquid before they are absorbed. The "gamma" rays discovered by Villard are probably analogous to $x$-rays and vibrations of the ether. They are not, as in the case of the first two, deviated by magnetic or electric fields, and are characterised by extraordinary penetrating power through matter. Thus chiefly the beta and gamma components concern us therapeutically. An observation of considerable importance lies in the fact that when beta rays meet an obstruction secondary gamma rays are set up, and conversely gamma rays produce secondary beta rays. Clinically it is difficult to estimate how much of the effect when the emanation penetrates tissues is due to the direct, and how much to the secondary rays therein produced, but as both types can be screened at will it is possible to project any intensity or combination of primary beta and gamma rays, and, allowing for the corresponding secondary ray production, we should be able, with improved technique, to apply at will a known beta and gamma concentration at different tissue levels. $X$-rays themselves, as far as is known, appear to be identical with gamma rays of relatively weak penetrating power, and, in making the outlines which follow, one has taken irradiation to signify the use of either beta-gamma radium emanation or the harder varieties of $x$-rays.

### Observations in the Past.

Let us now review some past observations on the effect produced by the emanation on malignant growths. A great number of classical experiments with Jensen's mouse sarcomas and other similar cancers, which could be inoculated from one animal to another, proved that not only did radium emanation retard or destroy these tumours *in situ*, but also that if the inoculated portion was first exposed to the emanation, it did not proliferate but underwent absorption. Webb, Morton, and Russ observed later that in such animals an immunity against subsequent implantation of non-irradiated tumours was developed, but that this immunity decreased to zero as exposure of the originally inoculated portion was prolonged. These and other experiments gave rise to much controversy at the time, but, in any case, they are of great interest in view of an immunity process being possibly brought into play, and as proving a disadvantage to exist in over exposure.

### Human Cancer.

With human growths in superficial positions, early stages and of small dimensions, there are by now a great number of well-established cures, and, to take an example from the early literature, Faure-Beaulieu and Dominici described an instance of growth springing from the gum of the upper jaw at the site of a canine tooth lost through dental caries. There was a history of three months before commencement of treatment and histological examination made before irradiation revealed a vascular myeloid sarcoma. After a series of exposures it was found that, at the end of four months, the tumour had regressed to a small fibrous scar. The observed changes were (*a*) metamorphosis of much of the protoplasm of the sarcoma cells into connective tissue fibres, (*b*) conversion of cytoplasm and nuclei of sarcomalous tissue into connective tissue cells, (*c*) obliteration of all large blood spaces.

In connection with carcinoma, Rubens-Duval and Dominici gave an instance of mammary growth. They consider that in favourable cases they have seen, destruction of a part of the malignant cells is followed by absorption of the rest exactly as suggested in mouse cancer; and we give the example as possibly connected with the absorption idea, as well as definite instance of tumour regres-

**Rays and the Cancer Problem**—*(continued)*.

sion. Screened rays were used for seventy hours on the growth, which was eighty square centimetres in area, ulcerated, markedly indurated, and adherent to subjacent muscle. Three months after this treatment the growth was reduced to half its size and the ulceration practically healed, but at this point an intercurrent disease—acute bronchitis—caused the unexpected death of the patient.

A careful microscopic examination was then possible and, as in the previous case, gradual absorption of malignant cells and replacement by connective tissue was confirmed.

Morson by imbedding in growths platinum tubes containing radium for periods of fifteen to twenty hours and removing portions of tissue at intervals, proved the progressive degeneration of all cancer cells directly exposed to the rays, and a little later Knox observed that this degeneration varied greatly in extent, as various concentrations of beta and gamma rays were allowed to act.

### Later Experiences.

Such experimental and clinical work led, as in all similar instances, to a rush of conclusions and optimism; but nowadays there is no doubt that we do not always obtain complete and uncomplicated results such as Dominici at first described. In simple ray treatment failure invariably arises with extensive growths, with deep secondary deposits, etc., but it is exactly such instances which often defeat measures exclusively surgical.

Already we have referred to projection of known beta-gamma concentrations, and it is in this connection that improvement will probably take place. At the moment experiments on the penetration of rays through metallic layers and their selective absorption at various levels has given invaluable indications of how the problem of concentrated local effect may be ultimately solved. Cancerous has much greater absorptive powers than normal tissue; it is relatively selective in its reception of the rays. Therefore it becomes feasible to assume, whatever strength of emanation be used, that more degeneration or inhibition will be produced on any cancerous tissue if normal and not malignant cells intervene between it and the source of the rays. At once the conclusion is suggested that if the main growth be removed as completely as possible by surgical means, radium emanation will become far more effective on deep-lying inoperable fragments and metastases. In this way a recurrence might be at least delayed if not prevented.

### Pre-Operative Application.

In the early experiments already quoted there is definite indication that destruction of a part of the cancer cells by irradiation may be sometimes followed by the absorption of the rest. It is true that this has been known to occur after mere partial surgical removal, but whatever theory or explanation we choose one sees a definite suggestion that in clinically operable or inoperable growths, the pre-operative use of radium should be both directly and indirectly beneficial. There is, too, the possibility that some form of acquired bodily immunity or absorbing power may be thus produced before operation is commenced.

If so, it makes the case the stronger, and should such ideas be substantiated the obvious indication is to irradiate tumours both before and after operation. Authorities state that there is never any fear that irritation and increased rate of tumour growth may result. Also, that experience has shown that the time of exposure needed, markedly to inhibit the growing activity of cancerous cells, is far below the periods known to have given rise to any complications in the normal tissues. Instances where this has occurred have been instances of gross and unnecessary over exposure.

### In the Future.

We must not expect to find a specific cure or an absolutely selective and otherwise harmless agent in either radium emanation or $x$-rays. The extent of both their power and their limitations must be realised. If the latter have given such methods a reputation of failure one should not too readily accept the conclusion until the experimental work is completed and more widely known. We would refer the reader to recent issues of the *New York Medical Journal* and *Medical Record*, where excellent reviews by American surgeons leave one with the impression that a correct combination of surgery with radium and $x$-ray therapy may still produce an efficient method of combating this dread disease. We hope soon to share their optimism.

